# Quercetin: Molecular Insights into Its Biological Roles

**DOI:** 10.3390/biom15030313

**Published:** 2025-02-20

**Authors:** Hye Joon Boo, Danbi Yoon, Yujeong Choi, Younghyun Kim, Jeong Seok Cha, Jiho Yoo

**Affiliations:** 1 College of Pharmacy, Chung-Ang University, Seoul 06974, Republic of Korea; 2 Research Institute of Pharmacy, Chung-Ang University, Seoul 06974, Republic of Korea

**Keywords:** quercetin, flavonoid, protein structure, molecular mechanism, structural biology, X-ray crystallography

## Abstract

Quercetin, a prevalent plant flavonoid, demonstrates many biological functions through its interaction with distinct protein targets. Recent structural investigations of protein–quercetin complexes have elucidated the molecular mechanism behind these actions. This paper presents a thorough structural analysis of experimentally established protein–quercetin complex structures published to date. The structure of the protein–quercetin complex elucidates the molecular mechanism by which quercetin influences protein function. These structures illustrate how quercetin’s chemical characteristics facilitate diverse modes of action by enabling particular interactions with the target protein. This structural knowledge provides the molecular foundation for comprehending quercetin’s biological roles and indicates avenues for future structural investigations of flavonoid–protein complexes, especially those with ambiguous molecular processes.

## 1. Introduction

Flavonoids are a diverse group of polyphenolic compounds widely distributed in plants, known for their significant biological activities. Among them, quercetin (3,3′,4′,5,7-pentahydroxyflavone) is a prevalent flavonoid found in nature, extensively present in vegetables, fruits, tea, and wine [1,2]. This naturally occurring chemical has been thoroughly investigated for its wide range of biological functions. Research has shown that quercetin plays significant roles in multiple cellular processes: it serves as a powerful antioxidant compared to other flavonoids by directly neutralizing reactive oxygen species [3], modulates inflammatory responses via cytokine production regulation [4], influences cell cycle progression [5], and impacts cellular energy metabolism [6]. Furthermore, quercetin has demonstrated the ability to regulate ion channel activity [7] and affect epigenetic alterations [8]. One of the primary reasons for quercetin’s importance is its ability to modulate multiple cellular pathways, including the NF-κB and MAPK signaling pathways, which play crucial roles in inflammation and oxidative stress [1,9]. Additionally, quercetin’s structural properties contribute to its high radical scavenging capacity and interaction with key enzymes involved in disease pathogenesis, further reinforcing its significance among flavonoids [3]. Given its diverse biological functions and therapeutic potential, quercetin is often considered the most important flavonoid in both dietary and medicinal contexts.

**Figure 1:**
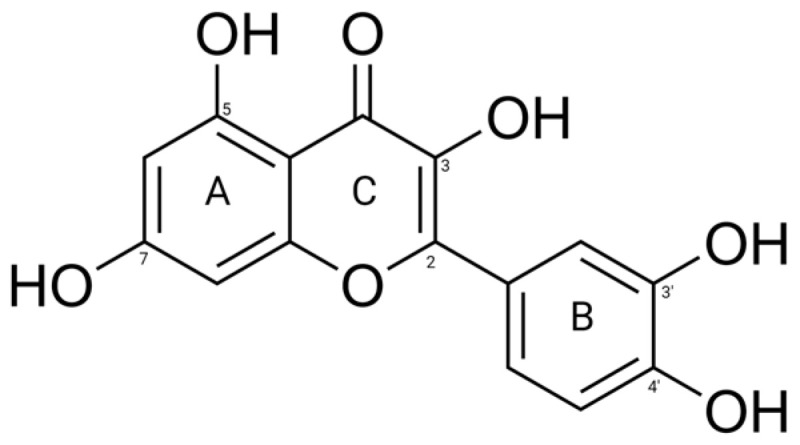
The chemical structure of quercetin. Each ring is labeled, and carbons that have a hydroxyl group are numbered separately.

As shown Figure 1, the molecular structure of quercetin has two aromatic rings (A and B) linked by a ring structure (C), including five hydroxyl groups at designated locations [10]. This distinctive structure allows quercetin to engage in multiple molecular interactions, such as hydrogen bonding and π-π stacking. Comprehending how these structural characteristics influence protein binding is essential for clarifying the molecular mechanisms that govern quercetin’s biological functions.

Recent breakthroughs in structural biology have provided exceptional insights into the molecular complexities of protein–quercetin interactions. These structural investigations have clarified the interaction of quercetin with many protein targets and its modulation of their functions. The availability of these high-resolution structures has generated new opportunities for understanding the molecular basis of flavonoid–protein recognition. This paper examines the structural characteristics of protein–quercetin complexes that have been empirically established. We examine the binding modalities, molecular mechanisms, and structural characteristics that allow quercetin to regulate protein function. By meticulously analyzing these structures, we seek to elucidate how quercetin exerts its biological effects at the molecular level. This review examines various protein families and illustrates distinct mechanisms of quercetin action. Through a collective analysis of these structures, we may discern universal principles of quercetin recognition by proteins, with distinct characteristics that facilitate selective target modulation.

Comprehending these structural features is crucial for elucidating quercetin’s biological functions and offers significant insights into the wider field of protein–flavonoid interactions. This knowledge will also inform future structural investigations of flavonoid–protein complexes whose molecular mechanisms are still to be clarified.

## 2. Structural Analysis of Protein–Quercetin Complexes

### 2.1. Xanthine Oxidase (XO)

Xanthine oxidase (XO) is a highly conserved enzyme containing molybdenum that catalyzes the final steps in purine degradation [11]. The enzyme serves various physiological functions, primarily facilitating the oxidation of hypoxanthine to xanthine and then to uric acid, which are the final two stages in purine nucleotide breakdown [12]. In these reactions, XO employs molecular oxygen as an electron acceptor, resulting in the formation of superoxide anion (O2•-) and hydrogen peroxide (H_2_O_2_) as byproducts. The capacity of XO to generate reactive oxygen species renders it a significant factor in cellular redox homeostasis [13]. Under standard physiological condition, XO activity is meticulously managed to sustain optimal uric acid levels and redox equilibrium [14]. However, in pathogenic situations, excessive activation of XO might result in the overproduction of uric acid and reactive oxygen species (ROS). Increased XO activity has been associated with several illness conditions: hyperuricemia and gout [14], ischemia–reperfusion injury [15], cardiovascular diseases [16], and inflammatory disorders [17].

Quercetin has been recognized as a powerful inhibitor of XO, demonstrating therapeutic potential in illnesses linked to increased XO activity. Biochemical research has shown that quercetin can significantly diminish XO-mediated ROS generation and uric acid synthesis [18]. Quercetin’s inhibitory action on XO is notably significant as it concurrently targets both facets of XO-mediated pathology: excessive uric acid production and oxidative stress. The dual inhibitory mechanism of quercetin renders it a compelling chemical for therapeutic applications aimed at XO-related clinical disorders [19].

XO operates as a homodimer, with each subunit comprising unique domains that contain various redox centers: the molybdenum cofactor (Mo-co), two dissimilar [2Fe-2S] clusters, and one FAD center [20]. The catalytic mechanism of XO entails a complex electron transfer chain orchestrated by redox centers. The process commences when the substrate (hypoxanthine or xanthine) associates with the molybdenum center via coordination with a Mo=S group. This interaction initiates the base-assisted deprotonation of the substrate’s hydroxyl group, subsequently leading to hydride transfer to the Mo=S group, which facilitates the two-electron reduction of Mo(VI) to Mo(IV) as the substrate undergoes oxidation [21]. The electrons produced from substrate oxidation subsequently enter a meticulously organized electron transport pathway. Initially, they are conveyed from the diminished molybdenum center to the proximal iron–sulfur cluster [Fe-S I], followed by transfer to the distal iron–sulfur cluster [Fe-S II]. The electrons subsequently transfer to the FAD core, where they produce hydrogen peroxide or superoxide [22].

**Figure 2:**
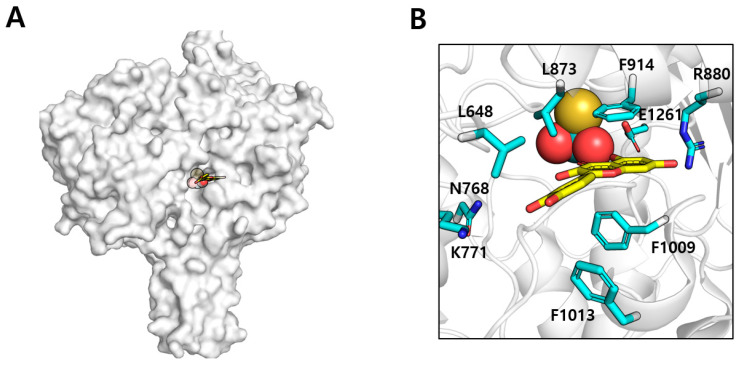
(**A**) Monomeric structure of XO bound with quercetin and Mo-co. Quercetin is modeled with sticks, and Mo-co is represented by small spheres. (**B**) The quercetin bind pocket. Quercetin is represented by a yellow stick, and residues that surrounded a quercetin are colored cyan. The thio-molybdenum ion and two oxygens that comprise the molybdenum cofactor are shown as a yellow sphere and red spheres, respectively.

The crystal structure of XO in complex with quercetin (PDB ID: 3NVY, Figure 2) demonstrates that quercetin associates within the constricted channel that is directed to the molybdenum core, the active region for physiological substrate binding [23]. The crystal structure elucidates numerous critical characteristics of the quercetin binding pocket in XO. A profound binding cavity is established by many structural components, forming a distinct pocket that houses quercetin. As shown in Figure 2, the pocket contains both hydrophobic and polar residues essential for substrate recognition and binding [23]. In this binding site, quercetin adopts a precise orientation that optimizes its interactions with the protein (Figure 2). The A- and C-rings of quercetin are situated deep within the hydrophobic cavity of the binding pocket, whereas the B-ring protrudes toward the pocket entrance [23].

The structural study of the XO–quercetin complex elucidates the molecular mechanism of enzyme inhibition. Kinetic investigations indicate that quercetin functions as a competitive inhibitor of XO, directly obstructing substrate binding [19]. As shown in Figure 2, the crystal structure clearly illustrates the molecular basis for this inhibition, revealing that quercetin occupies the same binding pocket as the physiological substrate hypoxanthine and xanthine [23]. The efficacy of quercetin’s inhibition is augmented by its capacity to establish particular contacts that obstruct substrate binding. The network of hydrogen bonds established with critical catalytic residues guarantees stable binding and efficient competition with the natural substrate. Among the various compounds that act as XO inhibitors, allopurinol is one of the most well known. Structural analysis of the allopurinol–XO complex reveals that the inhibitor binds at a site similar to that of quercetin, suggesting that quercetin may also function as an XO inhibitor in a comparable manner [24]. In conclusion, quercetin functions as an inhibitor of XO by directly attaching to the enzyme in a manner analogous to its substrate, xanthine, thereby inhibiting XO activity.

### 2.2. Phosphoinositide 3-Kinase (PI3K)

Phosphoinositide 3-kinase (PI3K) is a family of lipid kinases essential to cellular signaling networks. These enzymes catalyze the phosphorylation of phosphatidylinositol-4,5-bisphosphate (PIP2) at the 3-position of the inositol ring, yielding phosphatidylinositol-3,4,5-trisphosphate (PIP3), with ATP serving as the phosphate donor [25,26]. This reaction is a vital regulatory step in various physiological processes, since PIP3 serves as a second messenger that recruits and activates downstream effector proteins.

PI3K signaling governs various cellular activities vital for growth, survival, and metabolism. The process is initiated by several growth factors and hormones via receptor tyrosine kinases, resulting in the membrane recruitment and activation of PI3K [27]. Upon activation, PIP3 produced by PI3K initiates the activation of AKT/protein kinase B, which subsequently phosphorylates many downstream substrates associated with cell survival, proliferation, and metabolic control [28]. The dysregulation of PI3K signaling is commonly noted in numerous clinical situations, especially in cancer. Mutations in components of the PI3K pathway or its upstream regulators can result in persistent pathway activation, facilitating unregulated cell proliferation and survival [29]. The pivotal function of PI3K in cell signaling and illness has established it as a vital therapeutic target, prompting significant investigation into its structure and regulation [30].

**Figure 3:**
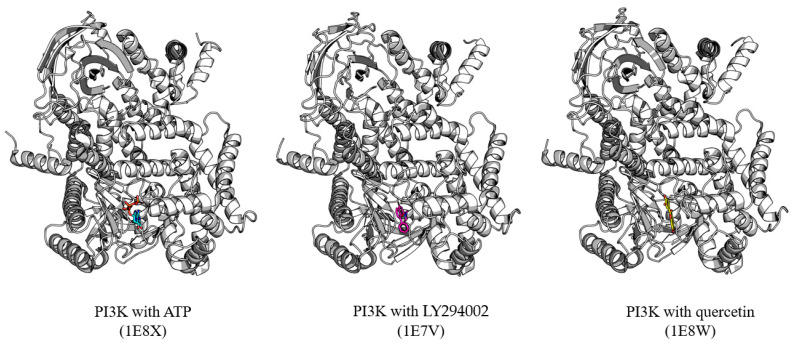
PI3K complex structure with ATP (**left**), LY294002 (**middle**), and quercetin (**right**). PDB IDs for each structure are labeled below.

PI3K is a heterodimeric enzyme consisting of a p110 catalytic subunit and a p85 regulatory subunit [31]. The p110 catalytic subunit comprises various domains: an N-terminal adaptor-binding domain (ABD), a Ras-binding domain (RBD), a C2 domain, a helical domain, and a C-terminal kinase domain. The kinase domain contains the ATP-binding site and the catalytic apparatus required for phosphate transfer [32]. The crystal structure of PI3Kγ (p110γ) complexed with quercetin (PDB ID: 1E8W) elucidates the molecular mechanism behind quercetin’s inhibition of PI3K activity [33]. Quercetin occupies the ATP-binding pocket within the kinase domain, where the adenine ring of ATP typically binds (Figure 3). The binding site is constituted by residues from the N- and C-terminal lobes of the kinase domain, resulting in a profound cleft typical of protein kinases. In the crystalline structure, quercetin assumes a distinct orientation within each binding space. In the binding configuration of PI3K and quercetin, quercetin seems to associate with the ATP binding site in a planar conformation; however, due to the incomplete orientation of the B-ring component, there exists the potential for quercetin to bind to PI3K in a conformation distinct from the planar form [33]. The binding of quercetin is reinforced by many unique molecular interactions within the ATP-binding pocket. The A- and C-rings of quercetin engage with the backbone between E880 and V882 of PI3K, analogous to the binding of adenine in ATP. However, the B-ring establishes an interaction with Lys833 of PI3K, similar to the interaction between LY294002 and PI3K, which is recognized as a PI3K inhibitor (Figure 3). Nevertheless, the interaction between Lys833 and LY294002 of PI3K occurs through the chromone moiety of LY294002, indicating that the binding mechanisms of quercetin and LY294002 are distinct [31].

The structural study of the PI3K–quercetin complex elucidates the molecular mechanism via which quercetin lowers PI3K activity [33]. Kinetic investigations indicate that quercetin functions as an ATP-competitive inhibitor of PI3K [34]. The crystal structure vividly illustrates the structural basis for this competitive inhibition, revealing quercetin filling the ATP-binding pocket and directly competing with ATP for binding (Figure 3). The mechanism by which quercetin inhibits PI3K encompasses numerous critical structural characteristics [31]. The planar chromone rings of quercetin occupy the same spatial region as the adenine base of ATP, thus obstructing ATP binding. The arrangement of quercetin’s A- and C-rings in the adenine-binding area, along with the B-ring’s expansion into the ribose-binding region, guarantees total obstruction of ATP access to the catalytic site. The efficacy of quercetin’s inhibition is augmented by its capacity to establish interactions that replicate those typically formed by ATP. The hydrogen connections established between the hydroxyl groups of quercetin and Val882 resemble the interactions commonly associated with the adenine base of ATP. Additionally, the hydrogen bond network with Asp841 stabilizes quercetin binding in a manner that prevents ATP binding. These interactions enhance binding affinity and ensure the correct orientation of quercetin within the catalytic pocket.

By blocking ATP binding, quercetin prevents the phosphate transfer reaction that is essential for PI3K’s catalytic function. This inhibition mechanism directly impacts PI3K’s ability to phosphorylate its substrate PIP2, thereby preventing the formation of the second messenger PIP3 [33]. The structural data explain quercetin’s observed IC50 value in the low micromolar range and provides a foundation for understanding the structure–activity relationships of flavonoid-based PI3K inhibitors [33].

### 2.3. HTH-Type Transcriptional Regulator TtgR (TtgR)

TtgR is a transcriptional regulator belonging to the TetR family of bacterial transcriptional repressors [35]. The modulation of TtgABC efflux pump expression in Pseudomonas putida is crucial for the ejection of antibiotics and other deleterious chemicals from bacterial cells. Under normal conditions, TtgR binds to certain operator sequences in the promoter region of the ttgABC operon, thereby suppressing the expression of the efflux pump genes.

The physiological role of TtgR centers on bacterial adaptation to environmental stresses, particularly the presence of toxic compounds. Suitable ligands interacting with TtgR induce conformational changes that reduce the protein’s affinity for DNA, leading to the downregulation of efflux pump genes [36]. This mechanism allows bacteria to rapidly respond to toxic agents by increasing the synthesis of efflux pumps. Understanding the molecular basis of TtgR’s interaction with various ligands, including plant-derived compounds like quercetin, clarifies bacterial multidrug resistance processes and the potential development of efflux pump inhibitors.

**Figure 4:**
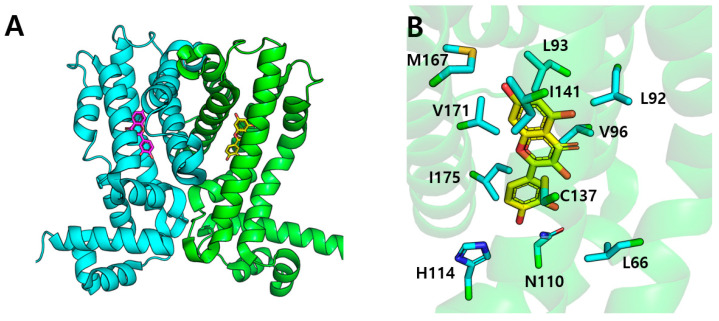
(**A**) Overall structure of TtgR dimer complex with two quercetin. Each monomer of TtgR is colored cyan and green. Quercetin that binds to each monomer is represented by magenta and yellow color. (**B**) Quercetin binding into TtgR. Residues that interacted with quercetin are labeled.

TtgR operates as a homodimer, with each monomer including nine α-helices (Figure 4A). The crystal structure of TtgR complexed with quercetin (PDB ID: 2UXH) demonstrates that each monomer has a ligand-binding domain (LBD) constituted of helices α4–α9 and a DNA-binding domain consisting of helices α1–α3 [36]. This structural configuration is characteristic of TetR family regulators, wherein ligand binding in the LBD influences DNA-binding activity. As shown at Figure 4A, the crystal structure indicates that quercetin associates within a substantial cavity of the ligand-binding domain of each monomer [36]. The crystal structure elucidates numerous critical characteristics of the quercetin binding region in TtgR. A profound binding cavity is established by the convergence of helices α4~α8, resulting in a distinct pocket that houses quercetin. The pocket contains both hydrophobic and polar residues essential for ligand recognition and binding. As shown in Figure 4B, the chromenone rings are positioned toward the upper region of the binding pocket, encircled by Leu92, Leu93 (α5), and Ile141 (α7), whereas the hydroxyphenyl ring is encompassed by Leu66 (α4), Val96 (α5), V171, and I175 (α8) [37]. Specifically, Asn110 and His114 from helix α6, Met89 and Leu92 from helix α5, and Asp172 from helix α8 are the principal contact sites within the binding pocket through hydrogen bonds [38]. In this binding site, quercetin adopts a precise orientation that optimizes its interactions with the protein. The A- and C-rings of quercetin are situated deep within the hydrophobic cavity of the binding pocket, whereas the B-ring makes a polar interaction with Asn110 and His114 for their binding (Figure 4B). The binding is reinforced by a distinct interaction: The hydroxyl groups of quercetin establish direct hydrogen bonds with His114 and Asn110, whereas Asp172 is involved in water-mediated hydrogen bonding networks. It is important to observe that Asn110 at the base of the ligand binding pocket engages with both positive (as in tetracycline) and negative (as in quercetin) charges through their side chains. Asn110 thus plays a crucial role in TtgR’s capacity to bind to both positively and negatively charged ligands [36].

The structural characterization of the TtgR–quercetin complex elucidates the molecular mechanism by which quercetin influences TtgR’s transcriptional regulatory activity [36]. TtgR generally operates as a repressor by attaching to particular operator sequences within the promoter region of the ttgABC efflux pump genes. When quercetin binds to TtgR, it initiates a sequence of conformational alterations that modify the protein’s DNA-binding characteristics.

The overall conformational alteration induced by quercetin resembles the binding of tetracycline to the TetR family [39]. Upon attaching to the binding pocket, quercetin interacts with α4 and α5, initiating a series of conformational alterations. The interaction of quercetin with Asn110 and His114 in α6 induces a conformational change that compels α4 to shift in the same direction due to van der Waals interactions (Figure 4B). The interactions with quercetin within the ligand-binding pocket cause a displacement of helix α6, which propagates throughout the protein structure, affecting the configuration of the DNA-binding helices α1–α3. The allosteric effect causes a reorientation of the helix–turn–helix patterns, making them unsuitable for DNA binding. Like tetracycline, quercetin obstructs the release of the binding cavity, preventing TtgR from re-binding to its target DNA.

The high-resolution crystal structure elucidates how quercetin’s interactions within the binding pocket induce these conformational alterations [36]. The network of hydrogen bonds established by the hydroxyl groups of quercetin with Asn 110 and His114, in conjunction with water-mediated interactions involving Asp172, stabilizes a distinct conformation of helix α6 (Figure 4B). This stabilization results in the repositioning of additional helices inside the ligand-binding domain, ultimately influencing the quaternary structure of the TtgR dimer [36]. The structural modifications cause TtgR to dissociate from its operator DNA, resulting in upregulation of efflux pump expression, perhaps as a defensive strategy against this phytochemical in bacteria.

### 2.4. (3R)-Hydroxyacyl-Acyl Carrier Protein Dehydratase (FabZ)

FabZ is a vital enzyme in the type II fatty acid synthesis (FAS II) pathway, needed for bacterial cell membrane formation [40]. This enzyme facilitates the dehydration of (3R)-hydroxyacyl-ACP to produce trans-2-enoyl-ACP, a crucial step in the elongation cycle of bacterial fatty acid biosynthesis. The FAS II pathway is exclusive to bacteria, plants, and parasites, differing from the type I fatty acid synthesis system seen in mammals. In bacteria, FabZ is pivotal in the production of both saturated and unsaturated fatty acids by catalyzing the dehydration process of β-hydroxyacyl-ACPs of varying chain lengths. The enzyme’s activity is crucial for preserving membrane lipid homeostasis and the viability of bacterial cells [41]. FabZ’s fundamental characteristics and its nonexistence in mammals render it a compelling target for the creation of antibacterial agents.

**Figure 5:**
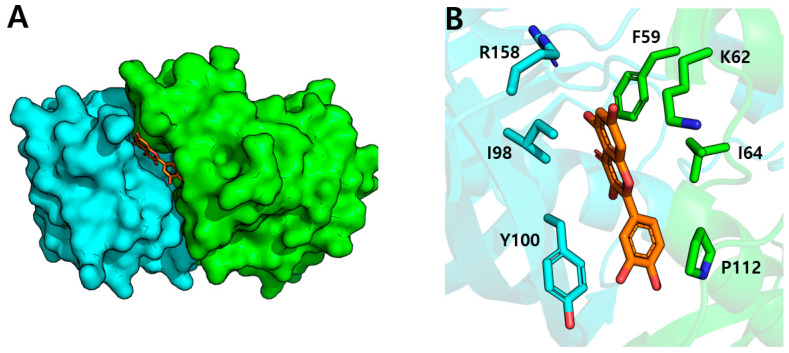
(**A**) Quercetin binding site of FabZ. Quercetin is bound to the dimer interface of FabZ dimer. (**B**) Residues comprising the quercetin binding site. Each residue for binding with quercetin is represented.

The importance of FabZ transcends its catalytic function. Its location at a critical junction in fatty acid biosynthesis renders it essential for modulating the ratio of saturated to unsaturated fatty acids, which is vital for bacterial membrane fluidity and response to environmental fluctuations. FabZ operates as a hexamer consisting of three asymmetric dimers, with each monomer featuring a catalytic domain [42]. As shown at Figure 5A, the crystal structure of FabZ complexed with quercetin (PDB ID: 3CF8) demonstrates that quercetin interacts at the interface of two neighboring monomers, occupying the substrate-binding tunnel typically utilized by the acyl chain of the natural substrate [43]. Each FabZ monomer comprises an elongated α-helix encircled by a curved β-sheet, analogous to a hot dog in a bun, which is a distinctive structural attribute of this enzyme family.

The crystal structure indicates that quercetin associates at the interface of two neighboring monomers, forming an extended binding pocket that is mostly hydrophobic yet has particular polar residues for ligand interaction (Figure 5B). The binding pocket is delineated by certain residues that establish the binding milieu: residues, including Phe59, Lys62, Ile64, and Pro112, constitute one side of the tunnel, whilst residues such as Ile98, Tyr100, and Arg158 comprise the opposing side [43].

The binding of quercetin entails numerous unique interactions with the protein. The flavonoid assumes a precise orientation within the binding tunnel, with its A- and C-rings situated deep within the hydrophobic pocket (Figure 5A). Multiple critical residues are involved in the recognition and binding of quercetin: Phe59, Ile111, and Pro112 from one monomer, along with Ile98, Tyr100, and Arg158 from the next monomer, establish direct hydrogen bonds with the hydroxyl groups of quercetin. Hydrogen bonds facilitated by water enhance binding stability, forming a comprehensive interaction network that accurately positions quercetin within the active site [43].

The structural characterization of the FabZ–quercetin complex offers comprehensive insights into the molecular mechanism of enzyme inhibition. Kinetic investigations indicate that quercetin functions as a competitive inhibitor of FabZ, directly obstructing substrate binding [44]. In Figure 5A, the crystal structure of FabZ in complex with quercetin reveals that quercetin binds at the dimer interface [43]. Interestingly, this binding site overlaps with the putative ACP binding site that was previously identified in the apo-FabZ structure [42]. This suggests that quercetin might interfere with substrate binding by occupying the region where the ACP moiety of the natural substrate would interact with the enzyme [42,43,44].

The inhibitory mechanism encompasses several critical elements shown by the structural study. Quercetin’s binding mode directly obstructs the enzyme’s catalytic mechanism by occupying the substrate-binding tunnel at the dimer interface. The crystal structure of FabZ in complex with quercetin reveals that quercetin binds at the dimer interface. This binding site coincides with the putative ACP binding site previously identified in P. aeruginosa FabZ [42]. The conserved catalytic residues (His58 in H. pylori FabZ, corresponding to His49 in P. aeruginosa FabZ) are located near the quercetin binding site. While quercetin does not directly interact with these catalytic residues, its binding at the putative ACP binding site suggests that it could interfere with substrate binding by preventing the access of the ACP-linked substrate to the catalytic residues. The efficacy of quercetin’s inhibition also is augmented by its capacity to form several specific contacts with both monomers constituting the active site. The network of hydrogen bonds established by the hydroxyl groups of quercetin with critical residues guarantees stable binding and efficient competition with the natural substrate. Moreover, the significant hydrophobic interactions involving Phe59, Ile64, Ile98, and Pro112 enhance the overall binding affinity and specificity of inhibition by quercetin [43,44]. Among some compounds that inhibit FabZ, the natural product juglone is one of the recognized. Structural analysis of the juglone–FabZ complex shows that the inhibitor occupies a binding site similar to that of quercetin, indicating that quercetin may also act as an inhibitor of FabZ in a similar fashion [45]. This structural configuration elucidates quercetin’s demonstrated inhibitory efficacy against FabZ and offers insights into the development of prospective antibacterial agents aimed at the bacterial fatty acid production pathway.

### 2.5. Transthyretin (TTR)

Transthyretin (TTR) is a homo-tetrameric protein essential for the transfer of thyroxine and vitamin A (via retinol-binding protein) in plasma and extracellular fluid [46]. Each TTR monomer comprises eight β-strands organized in a β-sandwich configuration, with four monomers assembling to create a central hydrophobic channel for thyroid hormone binding [46].

The biological importance of TTR transcends its role in transport. TTR is among the proteins implicated in amyloid disorders, characterized by protein misfolding and aggregation resulting in tissue dysfunction. Wild-type TTR can generate amyloid fibrils in senile systemic amyloidosis, but different TTR mutations are linked to familial amyloid polyneuropathy and cardiomyopathy [47]. The dissociation of TTR tetramers into monomers is a crucial phase in amyloid formation, rendering tetramer stabilization a significant therapeutic approach [48]. In recent years, TTR has been a prominent therapeutic target, especially with amyloid disorders. The discovery of tiny compounds that can stabilize the tetrameric structure of TTR and inhibit its dissociation into amyloidogenic monomers has emerged as a significant research focus [49].

**Figure 6:**
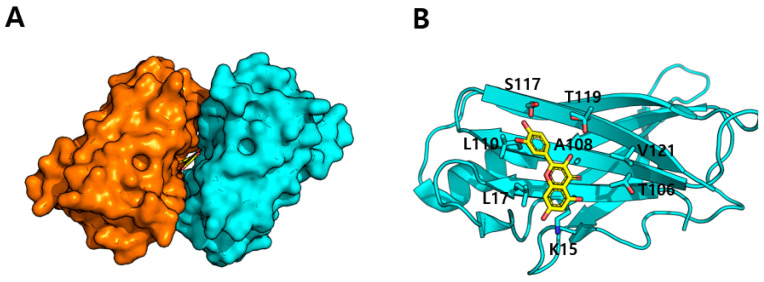
(**A**) Quercetin binding to TTR. Quercetin is bound at the dimer interface of TTR. (**B**) Residues comprising the quercetin binding site.

The crystal structure of TTR complex with quercetin (PDB ID: 4WNJ) demonstrates that quercetin associates with the thyroid hormone binding sites situated at the interface of two TTR dimers (Figure 6A) [50]. The structure reveals two identical quercetin binding sites per tetramer, symmetrically located at the dimer–dimer interfaces. The binding region is constituted by residues from both dimers, forming a deep pocket that is predominantly hydrophobic yet includes polar residues for ligand recognition [50]. The pocket is bordered by the side chains of numerous critical residues, including Lys15, Leu17, Ser117, and Thr119 from both monomers (Figure 6B). This configuration creates two symmetrical binding sites in the central channel of the TTR tetramer.

In the crystal structure, quercetin assumes a distinct orientation within each binding space. The B-rings of the flavonoid are situated deep within the inner region of the binding site, whereas the A-ring and C-ring protrude toward the entrance of the pocket. The binding of quercetin is reinforced by many unique molecular interactions inside the TTR binding pocket. The 4′-hydroxyl groups of the B-ring in quercetin establishes direct hydrogen bonds with Ser117, whereas its 5′-hydroxyl group participates in hydrogen bonding interactions with Thr119 (Figure 6B). The stability of the complex is additionally augmented by a network of hydrogen bonds, especially those associated with Thr119 and a hydroxyl group in C-ring of quercetin. The binding of quercetin is also additionally reinforced by its complementary conformation to the binding pocket and the establishment of many non-polar contacts (Figure 6B). The interactions collectively enhance the binding affinity and specificity of quercetin for TTR [50].

The structural analysis of the TTR–quercetin complex provides insights into how quercetin stabilizes the tetrameric structure of TTR and potentially prevents amyloid formation [50]. Unlike the previous proteins discussed, where quercetin acts as an inhibitor, in TTR, quercetin functions as a stabilizer of the native tetrameric state. The mechanism of TTR stabilization by quercetin involves several key structural features. When quercetin binds to the dimer–dimer interfaces of TTR, it establishes an extensive network of interactions that bridge between two dimers. This binding of quercetin strengthens the contacts between TTR dimers, thereby stabilizing the tetrameric assembly.

The effectiveness of quercetin’s ability to stabilize the tetrameric form of TTR is enhanced by its ability to simultaneously interact with residues from both dimers. The hydrogen bonds formed with Lys15 and Ser117 from opposing dimers act as molecular “staples” that help hold the tetramer together. Additionally, the hydrogen bond network involving Thr119 provides further stabilization [50]. These interactions, combined with the extensive van der Waals contacts, create a stable bridge between the dimers that helps prevent their dissociation (Figure 6B). This structural mechanism explains how quercetin can inhibit TTR amyloid formation by stabilizing the native tetrameric state, thereby preventing the formation of amyloidogenic monomers [51]. The binding of quercetin effectively increases the kinetic barrier for tetramer dissociation, which is the rate-limiting step in the amyloid formation process [52].

## 3. Conclusions and Future Perspectives

This review offers in-depth structural information on protein–quercetin complexes, elucidating the molecular mechanisms by which quercetin engages with various protein targets. This structural analysis has elucidated how this singular flavonoid molecule can regulate many protein functions via various binding modes and processes.

A comparative examination of these structures identifies multiple shared characteristics in quercetin binding. The planar configuration of quercetin facilitates its accommodation in diverse binding pockets, while its arrangement of hydroxyl groups permits the establishment of distinct hydrogen bonding networks. Quercetin functions as an inhibitor in XO, PI3K, FabZ, and TtgR by directly competing with endogenous substrates or ligands. In TTR, quercetin acts as a stabilizer of the native tetrameric state. Notwithstanding these common traits, each complex displays distinct qualities that influence certain biological results. In XO, quercetin obstructs substrate access to the molybdenum center; in PI3K, it occupies the ATP-binding site; in FabZ, it binds at the dimer interface; in TtgR, it promotes conformational alterations that influence DNA binding; and in TTR, it stabilizes the quaternary structure.

The structural data derived from these complexes have considerable implications for comprehending flavonoid–protein interactions in a broader context. The many binding types discovered indicate that quercetin’s biological effects stem from its capacity to conform to multiple protein binding sites while preserving unique interaction patterns [53]. The structural plasticity, along with the ability to establish diverse molecular contacts, elucidates quercetin’s aptitude to regulate multiple cellular targets.

Several significant areas await exploration. Initially, numerous anticipated quercetin–protein interactions remain devoid of structural elucidation. Secondly, the dynamic elements of these interactions, especially the protein flexibility [37], require additional examination. Ultimately, the structural insights obtained from this research may inform the development of more selective and powerful flavonoid-based drugs [54]. These structural investigations not only deepen our comprehension of quercetin’s molecular processes but also establish a basis for structure-based methodologies in the creation of flavonoid-inspired medicinal treatments. Subsequent research is expected to uncover further intricacies in flavonoid–protein interactions and may facilitate the creation of more potent molecules derived from the quercetin framework.

## Data Availability

Not applicable.
